# Saroglitazar in Non-alcoholic Fatty Liver Disease From Bench to Bedside: A Comprehensive Review and Sub-group Meta-Analysis

**DOI:** 10.7759/cureus.47493

**Published:** 2023-10-22

**Authors:** Akash Roy, Bikram Tewari, Suprabhat Giri, Mahesh Goenka

**Affiliations:** 1 Gastroenterology, Apollo Multispeciality Hospitals, Kolkata, IND; 2 Pharmacology, Sikkim Manipal Institute of Medical Sciences, Gangtok, IND; 3 Gastroenterology and Hepatology, Kalinga Institute of Medical Sciences, Bhubaneshwar, IND

**Keywords:** metabolic health, liver stiffness, diabetic dyslipedemia, nafld, saroglitazar

## Abstract

Non-alcoholic fatty liver disease (NAFLD) has become one of the most common causes of liver diseases globally, with a projected exponential rise. In contrast to the exponential rise in disease burden, there are limited options in the pharmacotherapeutic armamentarium against NAFLD. Saroglitazar belongs to the class of drugs known as peroxisome proliferator-activated receptor (PPAR) agonists, initially introduced for managing diabetic dyslipidemia. However, based on translational and clinical studies, it has been shown to be efficacious in NAFLD. It has been shown to modify key parameters in NAFLD, including reduction of transaminase levels, improvement in overall metabolic health, reduction of liver fat content, and improvement of liver stiffness and histology. Given the promising results, it has been made a part of society's guidelines in the therapeutic management of NAFLD. However, there remains a dearth of detailed reviews encompassing both pre-clinical and clinical data on the effectiveness of saroglitazar in NAFLD. In this review, we comprehensively review the pharmacology, pre-clinical data, and clinical studies on saroglitazar usage in NAFLD and conduct a subgroup meta-analysis of studies focussing on the impact of saroglitazar on liver stiffness changes.

## Introduction and background

Non-alcoholic fatty liver disease (NAFLD) refers to the presence of ≥5% steatosis in the liver in the absence of known causes of steatosis [1]. The entity encompasses diverse phenotypes, ranging from bland steatosis to steatohepatitis, advanced fibrosis, and cirrhosis [2]. The burden of NAFLD has seen an exponential increase globally, with current literature showing 25% of the global population being affected by NAFLD and a projected rise of 63% between 2015 and 2030 [1,3]. Contrasting to the meteoric rise in disease burden, the therapeutic armamentarium against NAFLD has, however, seen limited development [1]. While patients with non-alcoholic steatohepatitis (NASH) with stage 2 or higher fibrosis are candidates for liver-directed therapy, current treatment strategies are limited to risk factor mitigation, lifestyle and dietary adjustments, and, in specific cases, use of vitamin E and pioglitazone [4].

Saroglitazar belongs to the class of drugs known as peroxisome proliferator-activated receptors (PPARs) agonists, which was given marketing authorization in India in 2013 as an agent for the management of atherogenic diabetic dyslipidemia [5]. Based upon its unique mechanism of action, the drug showed efficacy in NAFLD and, based upon subsequent trials, was granted approval as an agent for NAFLD in India [6,7].

**Figure 1:**
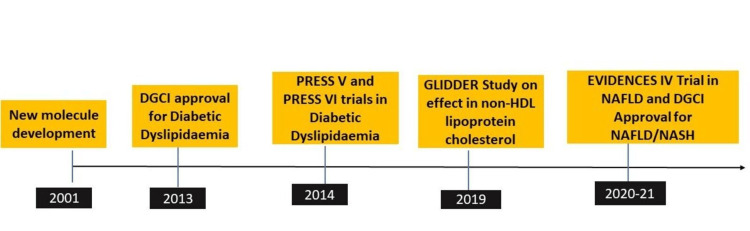
Showing timeline of saroglitazar development in NAFLD DCGI, Drug Controller General of India; NAFLD, Non-alcoholic fatty liver disease; NASH, Non-alcoholic steatohepatitis; HDL, High-density lipoprotein

A schematic representation of the development history of saroglitazar is shown in Figure 1.

However, there remains a paucity of literature that summarizes in a comprehensive manner the evidence for the use of this drug especially in the backdrop of recent landmark trials [5-7]. In this review, we aimed to review the available literature supporting the use of saroglitazar in patients with NAFLD with an attempt to incorporate both pre-clinical and clinical data and understand its usage in current-day practice. Additionally, we perform a subgroup meta-analysis on studies reporting changes in liver stiffness measurement (LSM) with saroglitazar.

## Review

Methodology

**Figure 2:**
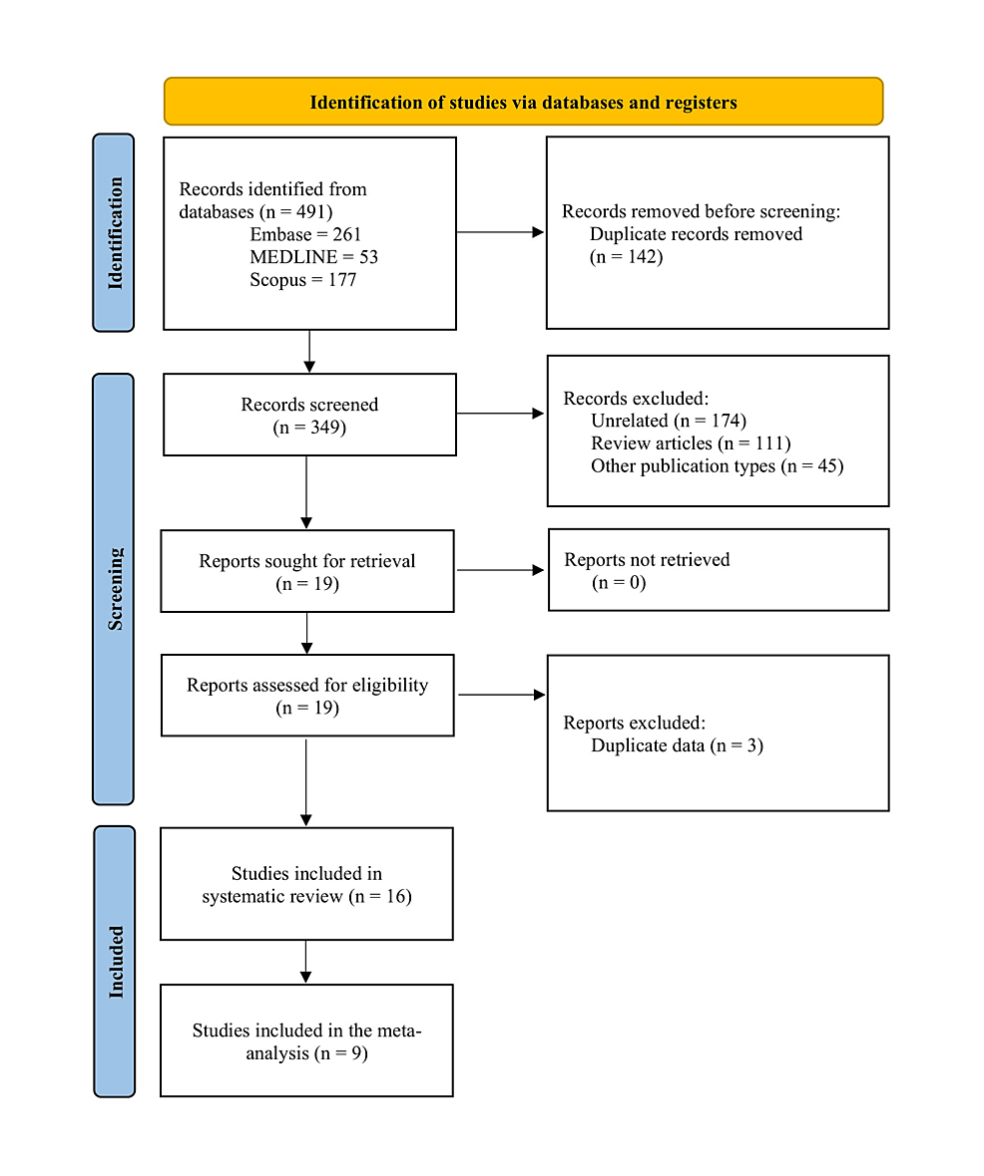
PRISMA flow diagram for the selection of studies on saroglitazar

We conducted this narrative review according to the guidelines and checklist provided by Green et al. [8]. Literature for this review was identified using the specific search term “Saroglitazar” in MEDLINE and EMBASE. All studies from the inception of the particular database to September 1, 2023, were searched. We reviewed all designs of articles (cohort studies, case-control studies, case series, case reports). Cohort studies, case-control studies, and case series were included, while case reports were excluded. The articles' language was restricted to English. After summarising the available literature, we performed a random-effects meta-analysis on studies reporting changes in LSM, a key component of efficacy acting as a surrogate for histological fibrosis reduction. We identified 491 papers (MEDLINE 53, EMBASE 261, and SCOPUS 177). Four pre-clinical studies and 12 clinical studies were identified for detailed review. Sub-group meta-analysis was carried out with nine studies for which relevant data were available. The PRISMA flow diagram is shown in Figure 2.

What are PPARs?

Prior to embarking on a detailed discussion on saroglitazar, it is imperative to understand the key receptors that form the crux of drug efficacy. PPARs are a group of nuclear receptors associated with the proliferation of peroxisomes. They are primarily involved in lipid and glucose metabolism with potential favorable effects on hepatic inflammation and fibrogenesis processes [9]. These receptors exist in three different isoforms (α, β/δ, and γ(two sub-isotypes γ-1 and γ-2)), which have variable tissue distributions and primary functions, while, specifically in the liver, these lead to a reduction of hepatic steatosis and improvement in inflammation and fibrosis [10]. Specifically, PPARα has a mechanistic basis for improving lipid metabolism by regulating lipid influx, fatty acid transport, and β-oxidation [9]. Additionally, it has been shown to reduce splanchnic inflammation and intestinal permeability. PPARβ/δ has anti-inflammatory properties exerted at the level of macrophages. PPARγ acts with potential regulatory roles in insulin sensitivity within the adipose tissue. Furthermore, PPARγ prevents hepatocyte stellate cell (HSC) activation, playing a key role in hepatic fibrosis pathways. Thus, working at various axes, the three PPAR isotypes act in different cells and organs, influencing different pathways and mechanisms involved in NASH and fibrosis progression [9].

Pharmacology of saroglitazar

As outlined before, saroglitazar is a PPAR agonist with predominant PPAR α and moderate PPAR γ activity. Hence, the molecule is essentially designed to induce the benefits of both fibrates and glitazone drugs. Previous molecules based upon similar mechanisms included muraglitazar, tesaglitazar, aleglitazar, and naveglitazar, all of which had safety concerns and, hence, were withdrawn from further studies [11]. The primary indication of the molecule was for dyslipidemia with favorable effects on glycaemic control, which led to its approval for “diabetic dyslipidemia.” This can be mechanistically explained by the reduction of TG mediated by the PPAR α agonism and improvement in insulin resistance and glycaemic control by PPAR γ agonism. Data from pharmacokinetic studies showed saroglitazar having good oral absorption (largely unaffected by food intake), with a median time to the peak plasma concentration of less than one hour (range 0.63-1 hour) and an average terminal half-life of 5.6 hours [12].

Evidence in diabetic dyslipidemia

In an extensive review, Kaul et al. provided insights into the potential benefits of saroglitazar [13]. The review of 18 studies spanning 5,824 patients (mean age 49.6-59.1 years, 22%-42% females) showed saroglitazar to consistently reduce triglyceride levels (45%-62%), total cholesterol levels (17%-26%), non-high-density lipoprotein cholesterol levels (21%-36%), low-density lipoprotein cholesterol levels (11%-27%), and glycosylated hemoglobin levels ( 0.7%-1.6%), leading to an increase in mean high-density lipoprotein cholesterol levels (up to 9%). The drug was extremely well-tolerated, with minor side effects reported as knee joint pain, chest discomfort, burning soles, and hypoglycemia [13].

Literature from pre-clinical studies in NAFLD

Multiple studies in pre-clinical mouse models have established the proof of concept for the efficacy of saroglitazar in NAFLD [14-17]. Saroglitazar at doses of 3 mg or 4 mg was shown to positively impact histology with improvement in hepatic steatosis, lobular inflammation, and ballooning. The study by Kumar et al. also showed significant improvements in fibrosis and NASH resolution in all cases [16]. Table 1 summarises the literature available from pre-clinical studies of saroglitazar in NAFLD/NASH.

**Table 1:** Preclinical animal model studies on saroglitazar in NAFLD CDAHFD, Choline-deficient L-amino acid defined high-fat diet; WDSW, Western diet sugar water, HOMA-IR, Homeostatic model assessment for insulin resistance, TG, Triglycerides; ALT, Alanine transaminase, NASH, Non-alcoholic steatohepatitis; HFHF, High fat, high fructose; SAR, Saroglitazar

Authors, Year	Model	Arms/Groups	Effects of Saroglitazar	Histological Effects
Akbari et al. (2021) [14]	Male Wistar rats fed with a high-fat emulsion	Saroglitazar (3 mg/kg), pioglitazone (30 m/kg), fenofibrate (100 mg/kg)and vehicle	Improvement in body weight, transaminases, leptin, and adiponectin decreased pro-inflammatory cytokines	Improvement in fatty appearance, lobular inflammation, hepatocellular ballooning decreased fibrosis
Kumar et al. (2020) [16]	DIAMOND mice fed with Western Diet Sugar Water (WDSW)	WDSW alone, WDSW plus pioglitazone(30mg/kg), WDSW plus saroglitazar (4mg/kg), vehicle control group	Improvement in weight, HOMA-IR, TG, total cholesterol, and ALT	Saroglitazar improved steatosis, lobular inflammation, hepatocellular ballooning, and fibrosis stage. NASH resolved in all mice receiving saroglitazar
Hasan et al. (2019) [15]	Female Wistar rats fed with standard chow diet and water ad libitum	Rats in group 1 (the control group) received saline (10 ml/kg/daily, oral gavage), while, in rats in group 2 (the HFE/LPS model group) and group 3 (the SAR-treated group), steatohepatitis was induced by the administration of HFE (10 ml/kg/day, oral gavage) and LPS (0.5 mg/kg/week)	Counteracted body weight gain and normalized liver function, glucose, (HOMA-IR) score, and lipid profile levels	Decrease in inflammation
Sarkar et al. (2021) [17]	C57BL/6 male mice on HFHF diet for four weeks	Saroglitazar (3 mg/kg/po), and Hepano - a formulation of five herbs (200 mg/kg/po)	Saroglitazar improved IR, obesity, reduced TG, and modulated phospholipids	None
Jain et al. (2018) [18]	HepG2 cells treated with palmitic acid (PA; 0.75 mM)	Saroglitazar (3 mg/kg), pioglitazone (25 mg/kg), and fenofibrate (100 mg/kg)	Significantly higher reduction of NAFLD activity score by saroglitazar	Antifibrotic effect of saroglitazar (4 mg/kg) observed in carbon tetrachloride-induced fibrosis model
Giri et al. (2023) [19]	Hepatocellular carcinoma induction in C57BL/6 mice by intraperitoneal injection of 25 mg/kg diethylnitrosamine (DEN) a	Saroglitazar (1 and 3 mg/kg) treatment for 27 weeks	All disease control animals showed hepatic tumors, which were absent in saroglitazar (3 mg/kg), indicating 100% prevention of tumorigenesis	None

Clinical studies of saroglitazar in NAFLD

Multiple clinical studies have subsequently emerged in patients with NAFLD analyzing the effects of saroglitazar [6,7,20-29]. The majority of the studies are from India and are single-center retrospective/prospective single-arm studies with variable follow-up, ranging from 12-52 weeks. Most studies from India have looked at biochemical improvements in transaminase levels and improvement in lipid profile parameters, while few studies have also looked at improvement in liver stiffness measurements and controlled attenuation parameter values [20,23-26]. Moreover, these data come from multicentric biopsy-proven studies and pooled individual data analysis of three multicentric cohorts [6,7,27]. Interestingly, one study also looked at the impact of saroglitazar on post-transplant NAFLD, reporting positive outcomes, and another abstract-only study reported similar results [30]. Two studies also included patients with compensated cirrhosis and reported no significant side effect concerns [25,26]. A detailed summary of the available evidence on saroglitazar based on different clinical studies is shown in Table 2.

**Table 2:** Clinical studies on saroglitazar NAFLD, Non-alcoholic fatty liver disease; NASH, Non-alcoholic steatohepatitis; ALT, Alanine transaminase; AST, aspartate transaminase; TG, Triglycerides; TC, Total cholesterol; HDL, High-density lipoprotein; LDL, Low-density lipoprotein; LSM, Liver stiffness measurement; SWE, Sheer wave elastography, NA, Not available, BMI, Body mass index, DM, diabetes mellitus, MRI-PDFF, Magnetic resonance imaging proton density fat fraction; CAP, Controlled attenuation parameter; ALP, Alkaline phosphatase; VLDL, Very low-density lipoprotein, Hba1c: Glycosylated hemoglobin; HOMA-IR, Homeostatic model assessment for insulin resistance

Authors, Year	Design	Arms	Population	Number of Patients	Follow-up	Key Demographics	Biochemical Changes	Safety
Padole et al. 2021 (India) [20]	Prospective	Single arm	NAFLD (No specifications)	91	12 weeks	Mean age=45 (18–66), 81% males, BMI 29.3 (23.6–42.2), ALT:48 (13–164), LSM:6.7 (3.6–13.1) 308 (249–400)	Outcomes divided into those with/without weight loss (5%). Weight Loss Group: Decrease in ALT, AST, CAP, and LSM (P<0.05) for all no weightloss group: Significant decrease in ALT, AST but not in LSM or CAP	NA
Jaiswal et al. 2021 (India) [21]	Retrospective	Single arm	Non-diabetic NAFLD	45	24 weeks	Mean age=46±8.20, 55% males, ALT 85.52±17.12, LSM:8.11±2.18, CAP365.84±56.22	Does not account for weight loss decrease in ALT, AST, CAP, and LSM (P<0.05) for all	NA
Roy et al. 2021 (India) [22]	Retrospective	Single arm	NAFLD with DM and dyslipidemia	10	36 weeks	Mean age=59.3 years, 70% males, BMI 25.21± 3.07, HbA1c 7.8±0.343, TG 298.2±35.75, ALT 64.7±15.56, SWE 1.837±0.0691	Significant decrease in all parameters (p<0.05 for all)	NA
Rajesh et al. 2021 (India) [23]	Prospective	Single	NAFLD with DM	85	12 weeks	Mean age 56.81 ±4.06 BMI 25.94 ±2.20 HBA1c 10.29 ±0.64 Triglycerides 359.89 ±5.46 HDL 49.20 ±3.08 SGPT 49.62 ±.31 LSM 9.68 ±0.30	Significant decrease in FBS, HBA1c, TC, TG, and SGPT, Mean decrease in LSM 3.61±3.98	No ADR
Goyal et al. 2020 (India) [24]	Prospective	Single	NAFLD with DD	107	24 weeks	Mean age 50.4 ± 12.3 BMI 28.8 ± 4.2 HBA1c 7.2 ± 0.65 TC 209.8 ± 62.4 Triglycerides 326.4 ± 98.5 HDL 38.2 ± 8.1 SGPT 94 (47–122) LSM 8.4 (7.1–9.3) CAP 335 (281–392)	Significant decrease in FBS, HBA1c, TC, TG, SGPT, SGOT, CAP, and LSM	Minor adverse events reported were fatigue in 2.8% (n=3), nausea in 1.9% (n=2), and dyspepsia 1.9% (n=2)
Siddiqui et al. 2020 (Multicentric, USA) [7]	Prospective	Double-blind placebo-controlled	Biopsy-proven NASH with NAS>4	16 paients Saro 2 mg:n=6 Saro 4 mg:n=7 Placebo n=3	24 weeks	Mean age 52±14; 85% of males rest not provided	Change in NAS was not statistically different with 4 mg (-1.9±1.57, p=0.60) when compared with saroglitazar 2 mg group (-1.5±0.84, p=0.77) and placebo (-1.3±0.58). Significant improvement in ballooning from 1.2±0.41 to 0.3±0.52 at week 24 with saroglitazar 2 mg and from 1.3±0.49 to 0.4±0.53 with saroglitazar 4 mg. Significant reductions in TG, TC, sd-LDL-C, and LDL-C	N=2 Not related to drug
Gawrieh et al. 2021 (Multicentric, USA) [6]	Prospective	Double-blind randomized	NAFLD established either by imaging (ultrasound, CT, or MRI) or liver biopsy showing NASH or simple steatosis and ALT ≥ 50 U/L	Saroglitazar 1 mg, n=26 group, saroglitazar 2 mg, n=25 group, saroglitazar 4 mg, n=27 group, and placebo n=28	16 weeks		The mean % ↓ ALT at week 16 was -45.8% (5.7) with saroglitazar 4 mg versus 3.4% with placebo. Significant ↓ in LFC [4.1%), HOMA-IR (-1.3), TG (-5.3 mg/dL) (p<0.05 for all). A mean weight gain of 1.5 kg was observed with saroglitazar 4 mg versus 0.3 kg with placebo (p>0.05).	Diarrhea n=3 cough n=3 Abdominal pain n=2 Bronchitis n=2
Mitra et al. 2020 (India) [25]	Prospective	Single	T2DM and NAFLD documented by ultrasonography of the abdomen	N=30	24 weeks	11 patients had fibrosis F3 grade (9.5-12.4 kPa), and 19 patients had a fibrosis F4 grade (≥12.5 kPa)	At the six-month changes were noted as (HbA1c) ↓ (8.14 ± 0.52% to 7.74 ± 0.53%) TG ↓ (179.4 ± 38.33 mg/dL to 112.33 ± 26.82 mg/dL) LSM↓ (13.933 ± 2.87 kPa to 8.503 ± 1.86 kPa ) P<0.05 for all	None
Chaudhuri et al. 2023 (India) [26]	Prospective	Single	Patients with NAFLD with elevated ALT levels along with liver stiffness value ≥6 kPa and/or liver steatosis CAP >290 dB/m	N=63	2-point follow-up analysis at 24 and 52 weeks	Mean age=49.1(±11.09), Mean BMI 27.2(±4,1), 46% DM, 27% dyslipedemic, mean LSM 8.5±3.9, mean CAP 320(±46) 11 patients had compensated cirrhosis	Significant ↓in LSM baseline: 11.03±7.19 kPa 24-week (9.29±6.39 kPa), 52-week 8.59±6.35 kPa. Significant ↓ in CAP, ALT, AST, HbA1c, LDL, TC, and TG levels	Pruritis in 1 Increase stool frequency in 1
Siddiqui et al. 2023 (Multicentric) [27]	Prospective	Pooled data analysis from multicentric phase II/III trials (USA, India, and Mexico)	Histologically proven NASH NAFLD or confirmed on the basis of imaging (ultrasound, computed tomography scan, or magnetic resonance imaging)	N=221 Saroglitazar 130 Placebo 91	16-24 weeks	Mean age=47.9±10.6 years, 56.1% females, mean BMI 30.9 ±5.3kg/m^2^ (36), 2% hypertensive, 32.6% DM, 21.7% on statins	Significant improvement in lipid parameters TC (–17 mg/dL, 95% CI, –24 to 9), TG (–45 mg/dL, 95% CI, –60 to 31), LDL (–8 mg/dL, 95% CI, –15 to –1), VLDL-C(–8 mg/dL, –14 to –3), and c sdLDL-C (–10 mg/dL, –17 to –2)	NA
Siddiqui et al. 2023 (USA) [28]	Prospective	Phase 2, single-arm study	Post liver transplant NAFLD NAFLD defined as CAP ≥264 dB/m. Primary endpoint: Liver fat reduction on MRI-PDFF	N=15	24 weeks	Mean age=58±12 years, mean BMI 37.4±7.4 kg/m^2^, DM: 26%, dyslipedemia 26%, hypertension 93%	Significant ↓ in MRI-PDFF (10.3±10.5% at baseline to 8.1±7.6%). A relative 30% reduction from the baseline MRI-PDFF value was noted in 47% of patients. Reduction ALP emerged as a predictor of PDFF response	Fluctuations in eGFR in two patients, one splanchnic vein thrombosis not related to drug
Hajare et al. 2019 (India) [29]	Prospective	Single arm	NAFLD and dyslipidemia with or without type 2 diabetes mellitus	N=52	52 weeks	Mean age=45.88±11.66 years, 71.15% males, 28.8% DM, 17.3% hypertensive, mean BMI 27.84±5.97 kg/m^2^	Significant ↓ALT (p<0.001), AST, (p<0.001), TG (p<0.001), LSM ↓12.33±9.99 to 9.62±4.53 9, p=0.01)	NA

Subgroup meta-analysis

**Figure 3:**
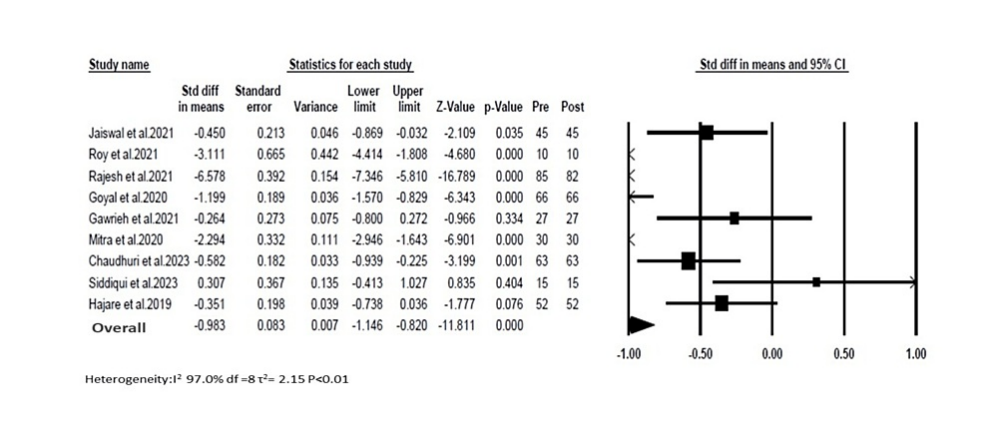
Showing the forest plot for random effects meta-analysis for liver stiffness measurement changes with saroglitazar in clinical studies

We conducted a subgroup meta-analysis (random effect) on studies that reported changes in LSM pre- and post-therapy with saroglitazar. Ten studies reported changes in LSM. We excluded the study by Padole et al. [20] as it did not provide overall LSM changes and subdivided based on weight changes [18]. We observed high heterogeneity among the studies (I2=97%). The overall pooled estimate for standard differences in means of LSM reduction was -0.98 (95% CI=1.1 to -0.8; Figure 3).

Discussion

Despite the epidemic proportions of NAFLD and consequent health implications, there remains a paucity of clinically and histologically effective drugs in the management of NAFLD [3]. Saroglitazar, based on its mechanism of action, is an effective molecule targeting key pathophysiological pathways and has a concomitant effect on atherogenic dyslipidemia. Our current review, transitioning from pre-clinical studies to real-world studies and randomized trials, indicates the effectiveness of saroglitazar on biochemical parameters, fat fraction reduction, and improvements in liver stiffness and histology. However, there remains a paucity of large placebo-controlled trials based on histology or its predicted surrogate markers such as MRi-PDFF fat fraction reduction [31].

A recent systematic review of saroglitazar, including 10 studies, showed overall pooled reductions in ALT, AST, glycated hemoglobin, total cholesterol, and triglyceride [32]. LSM changes in eight studies reported similar results, as shown by our analysis corroborating reductions in LSM, albeit with significant heterogeneity (99%). However, it needs to be borne in mind that most of the studies are single-center, single-arm studies originating out of India, and, hence, it mandates more extensive randomized trials from multiple centers.

The beneficial effects of saroglitazar potentially transcend beyond hepatic parameters as the drug has been used for a substantial period in atherogenic dyslipidemia. This is further reflected in the pooled analysis of NAFLD patients from three multicentric studies showing significant beneficial effects on lipid biomarkers and indicating cardiovascular benefits [27]. Safety has been an important point of concern for any drugs in the pipeline for NAFLD. Based on data from pre-clinical and clinical studies, the molecule appears to be safe and extremely well-tolerated. Of interest is the limited proportion of patients with compensated cirrhosis in two studies wherein no safety concerns were reported [25-26].

Our review has key strengths, the most important of which is for the first time we have collated evidence from both pre-clinical and clinical studies. We assessed, in a subgroup meta-analysis, the effect of the dug on LSM, which is one of the key factors that determine drug efficacy and serves as an excellent surrogate of fibrosis. The review has certain limitations, of which an important aspect is we did not analyze other markers such as ALT, fat fraction reduction, or change in metabolic parameters.

## Conclusions

In this review, we summarize for the first time the overall evidence of saroglitazar in NAFLD spanning across pharmacology, clinical, and pre-clinical studies. Saroglitazar, as a molecule, is safe in patients with NAFLD and, based on currently available literature, shows improvements in transaminase levels, metabolic parameters, liver fat content, and liver stiffness. However, larger multicentric biopsy-proven or adequate surrogates of biopsy-based studies are needed to promote global acceptance and approval.
